# *In silico* structural-functional characterization of three differentially expressed resistance gene analogs identified in *Dalbergia sissoo* against dieback disease reveals their role in immune response regulation

**DOI:** 10.3389/fpls.2023.1134806

**Published:** 2023-10-16

**Authors:** Siddra Ijaz, Imran Ul Haq, Hafiza Arooj Razzaq, Bukhtawer Nasir, Hayssam M. Ali, Sukhwinder Kaur

**Affiliations:** ^1^ Centre of Agricultural Biochemistry and Biotechnology (CABB), University of Agriculture, Faisalabad, Pakistan; ^2^ Department of Plant Pathology, University of Agriculture, Faisalabad, Pakistan; ^3^ Department of Botany and Microbiology, College of Science, King Saud University, Riyadh, Saudi Arabia; ^4^ Department of Plant Pathology, University of California Davis, Davis, CA, United States

**Keywords:** nucleotide-binding site, leucine-rich repeats, protein fingerprints, homology modeling, *Dalbergia sissoo*

## Abstract

Plant immunity includes enemy recognition, signal transduction, and defensive response against pathogens. We experimented to identify the genes that contribute resistance against dieback disease to *Dalbergia sissoo*, an economically important timber tree. In this study, we investigated the role of three differentially expressed genes identified in the dieback-induced transcriptome in *Dalbergia sissoo.* The transcriptome was probed using DOP-rtPCR analysis. The identified RGAs were characterized *in silico* as the contributors of disease resistance that switch on under dieback stress. Their predicted fingerprints revealed involvement in stress response. Ds-DbRCaG-02-Rga.a, Ds-DbRCaG-04-Rga.b, and Ds-DbRCaG-06-Rga.c showed structural homology with the Transthyretin-52 domain, EAL associated YkuI_C domain, and Src homology-3 domain respectively, which are the attributes of signaling proteins possessing a role in regulating immune responses in plants. Based on *in-silico* structural and functional characterization, they were predicted to have a role in immune response regulation in *D. sissoo.*

## 1 Introduction

*Dalbergia sissoo* (shisham) is a perennial tree species natively found on the Asian subcontinent and was introduced to other world regions a couple of decades ago. This tree has excellent value in forestry, agroforestry, horticulture, medicine, and the wood industry (Ijaz et al., 2019) but its rapid population decline limits its use in production. Shisham decline (dieback) was first reported in India and Bangladesh, and in 1998 it was declared an epidemic in Pakistan. Different phytopathogens are reported for their involvement in this disease (Ijaz et al., 2018; Ijaz and Haq, 2021).

To resist phytopathogen infection, plants have developed complex defense mechanisms. These mechanisms are a collection of several biochemical and physiological alterations in plants. The first level includes pathogen recognition, anti-pathogenic protein production, interruption of pathogen infection structures, and enzymatic cell wall reinforcement (Çetinel et al., 2022). If a pathogen overcomes it, then the second level initiates. This level includes resistance (R) genes or their product and starts a molecular cascade of signal transductions in response to the attack (Ijaz et al., 2022). The controlling signals include kinases, phytoalexin, peroxidases, reactive oxygen species, and guanine nucleotide-binding proteins (Zhong et al., 2019). R genes can be divided into four classes on their structural basis; class-I encodes ser-thr-kinases involved signaling network, and class-II encodes transmembrane receptor proteins with leucine-rich repeat (LRR) domains. However, class-III encodes receptor-like kinases and combines the properties of the first two classes, and class-IV encodes proteins of nucleotide-binding site-leucine-rich repeats (NBS-LRR) domains (Nasir et al., 2021).

Plant RGAs (resistance gene analogs) are the putative or candidate R genes featured for their conserved structure and are involved explicitly in pathogen resistance to plants. They include NBS-LRR, receptor-like proteins, and kinases. The best-known class is NBS-LRR, classified based on the presence and absence of the Toll/Interleukin-1 Receptor (TIR) domain at the N-terminus. The non-TIRs are attributed to the coiled-coil (CC) domain and the less common leucine zipper (LZ) domain (Babar et al., 2021). The NBS domain is approximately 300 amino acids in length with four subdomains, kinase 1a (P-loop), kinase 2, kinase 3a, and a hydrophobic GLPAL, which are involved in ATP/GTP hydrolyses and work as molecular switches in signal transduction network (Nasir et al., 2023). The LRR domain has approximately 10-40 leucine-rich-repeat motifs specified for pathogen recognition (Cannon et al., 2002). Their conserved sequence features are used to design degenerate primers to identify the RGAs with the NBS domain or RGAs with the LRR domain. Moreover, computational biology eases the identification of thousands of RGAs after plant genome sequencing. Therefore, several studies on genome-wide probing and identification of RGAs in many plant species have been done, including *Arabidopsis thaliana* (Tan et al., 2007), *Oryza sativa* (Zhou et al., 2004), *Medicago truncatula* (Ameline-Torregrosa et al., 2008), *Populus trichocarpa* (Kohler et al., 2008), *Vitis vinifera* (Yang et al., 2008), *Cucumis sativus* (Huang et al., 2009), *Brassica rapa* (Mun et al., 2009), *Carica papaya* (Porter et al., 2009), *Sorghum bicolor* (Paterson et al., 2009), *Brachypodium distachyon* (Li et al., 2010), and *Zea mays* (Cheng et al., 2012). The present study was carried out under CAS-PARB project number 952. This study was based on the identification of a dieback disease-resistant *D. sissoo* germplasm. In plants, diseases cannot be entirely eliminated, and the devastating situation is overcome through prevention and replacement. The replacement of diseased plant material with resistant sources is the best solution for disease management. Therefore this study was conducted to identify the resistant plant material of *D. sissoo* and the genes contributing to resistance to dieback disease in this tree species. Using a transcriptomic technique in this study, we identified genetic elements with differential expression under dieback challenge in resistant sources of *D*. *sissoo*. Subsequently, we characterized them using computational biology and identified their role in disease resistance.

## 2 Materials and methods

### 2.1 Primer designing

The NBS region of the NBS-LRR (Nucleotide Binding Site Leucine-Rich Repeats) class of *R* genes was used to probe the transcriptome of *D. sissoo* to identify the resistance gene sequence(s) against dieback disease. The NBS region of the NBS-LRR domain consists of conserved domains, such as kinase P-loop, kinase-2, kinase-3A, and hydrophobic GLPL motifs. Based on the NBS domain, degenerate primers were designed and synthesized to amplify resistance gene analogs (RGAs) against the dieback disease of *D. sissoo* (**Table S1**). Two primer sets were designed with the support of literature. One set from the 5’ P-loop domain was the forward primers, and the other from the 3’ GLPLA domain was the reverse primers.

### 2.2 Experimental site and plant material collection

A survey across Pakistan was conducted during 2017-2018 to collect healthy germplasm of *D. sissoo*. Branch cuttings of healthy plants were taken and macropropagated in the greenhouse of the Fungal Molecular Biology Laboratory, Department of Plant Pathology, University of Agriculture Faisalabad, Pakistan. For nursery raising, the cuttings were planted in polythene bags filled with a mixture of soil and sand (3:1 ratio) (Ijaz et al., 2019).

### 2.3 Plant inoculation

Macropropagated tagged material of *D. sissoo* was inoculated with *Ceartocystis dalbergicans* (Haq et al., 2023). Spore resuspension was adjusted to 1 × 10^11^ spores/ml for inoculations. Most plants died within a month post-inoculation. Several died in 2 months and we re-inoculated the remaining plants (with fewer or no disease symptoms) 3 months post-inoculation. The plants that still showed no symptoms after 6 months were tagged as resistant sources and probed for identifying resistance genes.

### 2.4 RNA isolation

Plants showing resistance under the dieback challenge were subjected to RNA isolation. The sampled leaves were immediately kept in liquid nitrogen prior to the RNA isolation procedure. Total RNA was isolated using the GeneJET Plant RNA Purification Mini Kit (Thermo Scientific, USA) following the manufacturer’s protocol. The dried pellet was dissolved in nuclease-free water and immediately used for downstream applications.

### 2.5 Transcriptome analysis

The extracted RNA of each sample was quantified on a UV-visible NANODROP (8000 Spectrophotometer, Thermo SCIENTIFIC), and quality was assessed through gel electrophoresis. The RNA samples were subjected to cDNA synthesis. First-strand cDNA was synthesized using the RevertAid First Strand cDNA Synthesis Kit (Thermo Scientific, USA), following the manufacturer’s protocol. Degenerate oligonucleotide primed-reverse transcriptase PCR (DOP-rtPCR) was performed to amplify putative disease resistance genes or RGAs (resistance gene analogs) from the transcriptome of *D. sissoo* under dieback stress. The reaction conditions for the DOP-rtPCR were optimized. Amplicons were resolved on 1.2% agarose gel and visualized under UV on the gel documentation system (Bio-Rad, USA). The required bands were cut and eluted from gel using a gel purification kit (FavorPrep) and sequenced on the Sanger platform (Eurofins Genomics, USA). The generated sequences were characterized *in silico*. The generated sequences of identified RGAs were translated using the Expert Protein Analysis System (ExPASy) translate tool.

### 2.6 Scanning of protein motifs

The translated proteins were scanned for structural motifs from the PROSITE collection of motifs. The ScanProsite web tool from ExPASy (https://prosite.expasy.org/scanprosite/) was used for this purpose.

### 2.7 Subcellular localization and physicochemical properties

The online web servers CELLO v. 2.5 (Yu et al., 2006) and SignalP 5.0 (Armenteros et al., 2019) were used to predict the subcellular localization of protein or signal peptides. An online ExPASy tool, ProtParam (Gasteiger et al., 2005), was used for computing the physicochemical parameters.

### 2.8 Identification of fingerprints and conserved domain

To identify conserved domains in the identified RGAs of *D. sissoo*, online tools from NCBI, CD-search (Lu et al., 2019), and CDART (Geer et al., 2002) were used. Similarly, protein fingerprints were scanned using the PRINTS database (Attwood et al., 1994).

### 2.9 Protein modeling

The secondary structures of deduced amino acid sequences were predicted and annotated through SOPMA (self-optimized prediction from multiple alignments) (Geourjon and Deleage, 1995) and PSIPRED 4.0 (Jones, 1999) online servers. In homology modeling analysis, SWISS-MODEL (Waterhouse et al., 2018) and PHYRE2 (Protein Homology/analogy Recognition Engine, Version 2.0) (Kelley et al., 2015) were employed. The template selection was based on significant score values. Their generated PDB files were used in PyMOL v. 2.4.0 for 3-D protein imaging. Ramachandran plots were generated through PROCHECK (Laskowski et al., 1993) to determine model quality. TM-align (Zhang and Skolnick, 2005) was employed for model superimposition.

## 3 Results

**Figure 1:**
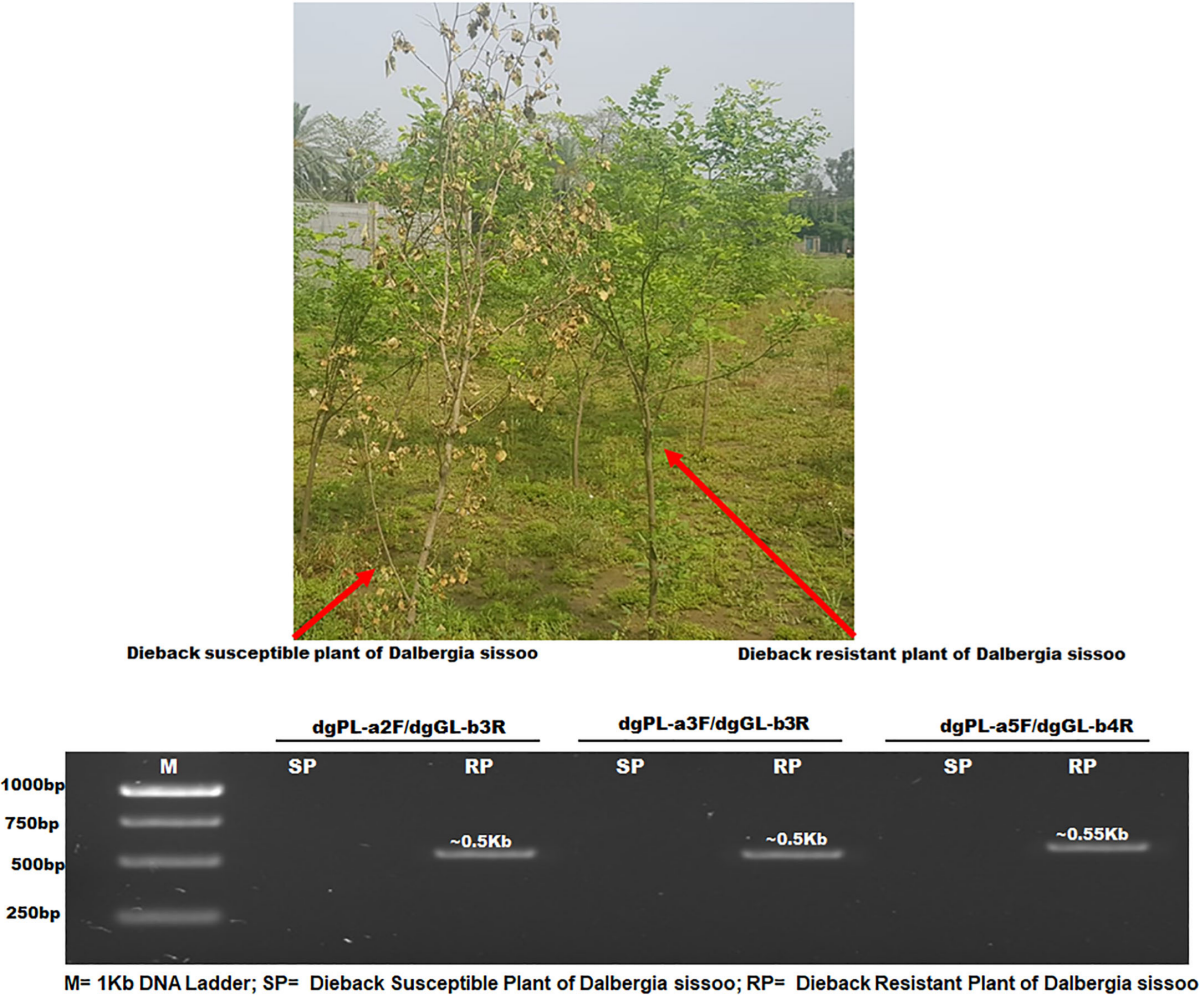
PCR analysis of dieback-resistant and susceptible plants of *D. sissoo*. M= 1Kb DNA Ladder; SP= Dieback Susceptible Plant of *D. sissoo*; RP= Dieback Resistant Plant of *D. sissoo.*.

DOP-rtPCR analysis was performed to identify RGAs expressed under dieback challenge in resistant sources of *D. sissoo* germplasm (**Figure 1**). The amplicons of dgPL-a2F/dgGL-b3R, dgPL-a3F/dgGL-b3R, and dgPL-a5F/dgGL-b4R (**Supplementary Table 1**) were sequenced. The translated protein sequences showed homology using Blastp to putative disease and stress resistance proteins. These RGAs were named Ds-DbRCaG-02-Rga.a (GenBank accession # MT946503), Ds-DbRCaG-04-Rga.b (GenBank accession # OP455119), and Ds-DbRCaG-06-Rga.c (GenBank accession # OQ552560).

### 3.1 Protein motif identification

Five different motifs were identified in the RGAs of *D. sissoo*. These five motifs were found with different signatures (**Table 1**). A protein kinase phosphorylation site was observed in the recovered RGAs.

**Table 1:** Identified conserved PROSITE motifs among *D. sissoo* RGAs recovered under dieback challenge.

RGAs	Scanned motifs	Motif signature
Ds-DbRCaG-02-Rga.a	N-myristoylation site	GSSSGD
	Casein kinase II phosphorylation site	SSGD
	Phenyl group binding site (CAAX box)	CVLR
Ds-DbRCaG-04-Rga.b	N-Glycosylation site	NGTR
	Protein kinase C phosphorylation site	SYR SSR
Ds-DbRCaG-06-Rga.c	N-myristoylation site	GTAPCF GLLRSI GVWVGT GISLSL GLESSS

### 3.2 Subcellular localization and physicochemical properties of *D. sissoo* RGA proteins

Subcellular localization prediction analysis was made in CELLO v 2.5. It confirmed their role as a subcellular entity. The physical and chemical properties of the deduced *D. sissoo* RGA proteins were also computed. These included amino acid composition, molecular formula, molecular weight (Mw), theoretical pI (isoionic point), aliphatic index, instability index (II), and the grand average of hydropathicity (GRAVY) of the deduced amino acids (**Supplementary Table 2**). The biochemical and molecular functioning of proteins relies on these properties. Amino acid composition confirmed the low abundance of methionine (0.9-1.7%), tryptophan (1.6-1.8%), and aspartate (1.6-3.3%), while there was a higher abundance of leucine (10.5-15.3%), proline (10.5-13.3%), serine (11.9-15.0%), and arginine (8.3-16.7%) in the deduced amino acid sequences. The isoionic or isoelectric point of a protein measures the pH at which its net charge becomes zero or neutral due to an ionizable group, i.e., aspartic acid, arginine, cysteine, glutamic acid, histidine, tyrosine, and lysine. All of these predicted proteins showed the basic range of isoelectric points. While the aliphatic index is based on hydrophobic and non-polar side chain residues, i.e., leucine, isoleucine, valine, and alanine, its high value indicates that the protein is thermally stable. The aliphatic index of the deduced amino acid sequences of the identified RGAs predicted that these proteins are thermally stable (**Supplementary Table 2**). Stable protein instability index (II) values should be 40 or lower. According to this prediction, the Ds-DbRCaG-02-Rga.a protein was categorized as stable. GRAVY measures the addition of the hydropathy values of the side-chain amino acids, while the sequence length is taken as a divisor. A higher GRAVY score (+ve) for proteins indicates an excellent hydrophobic nature. Among the identified RGAs, DbRCaG-02-Rga.a was predicted to have a hydrophobic nature, scoring a 0.675 value.

### 3.3 Identification of fingerprints and conserved domain

The translated proteins of the *D. sissoo* RGAs had no conserved domains upon searching through CDART and CD programs. The FPScan predicted the protein fingerprints of the translated amino acid sequences of these RGAs. The translated protein sequence of Ds-DbRCaG-02-Rga.a was predicted with an ELONGATNFCT (elongation factor signature) fingerprint. The protein synthesis elongation factor (EF-Tu) binds to the GTP molecule during the translation process and is now thought to be involved in plant stress response. The ELONGATNFCT is a five-element fingerprint that dispenses a signature to GTP-binding elongation factors. These five motifs were derived from the C-terminal site of the initial alignment of 12 elongation factors. The translated protein sequence of Ds-DbRCaG-01-Rga.a had shown homology with motif-2 and motif-4 in the ELONGATNFCT signature.

The translated protein sequence of Ds-DbRCaG-04-Rga.b was predicted with a VDCCGAMMA6 (voltage-dependent Ca^2+^ channel gamma 6) fingerprint. Calcium ion (Ca^2+^) concentration is an essential indicator of signal transduction during plant growth, development, and stress. These consist of four subunits: beta subunit (intracellular); gamma subunit (transmembrane); the cleaved product of the same gene, alpha-2; and delta subunit (Witcher et al., 1993). The VDCCGAMMA6 is a five-element fingerprint that provides signature sequences for the voltage-dependent calcium channel gamma6 subunit. These five motifs were derived from the initial alignment of four sequences. Motifs 1 and 2 reside in the cytoplasmic N-terminal site, motifs 3 and 4 in the first extracellular loop, while motif-5 covers the transmembrane domain-4, including the intracellular segment C-terminal region. The translated protein sequence of Ds-DbRCaG-01-Rga.b had shown homology with motif-1 and motif-3 in the VDCCGAMMA6 signature.

The translated protein sequence of Ds-DbRCaG-06-Rga.c was predicted with three fingerprint sequences. TNFACTORR13C provides signature sequences for necrosis or apoptosis as an activated host defense (Chu, 2013). The TNFACTORR13C is a three-element fingerprint derived from the initial alignment of three sequences based on the conserved region. The extracellular N-terminus region is covered by motif 1, while motif 2 spans at the N terminus and a transmembrane region. However, motif 3 resides in the C-terminus intracellular region. The translated protein sequence of Ds-DbRCaG-06-Rga.c predicted homology to motif-2 and motif-3 signature sequences. The second fingerprint is PROTEASOME provides a signature for proteasome subunits. The accumulation of proteasomes during stress is evidence of enhancing the gene expression of this protein complex. The proteasome first converted damaged protein into a short peptide chain and later to single amino acids used as building blocks of other proteins. The PROTEASOME is a four-element fingerprint derived from the initial alignment of six sequences based on conserved region.

The translated protein sequence of Ds-DbRCaG-06-Rga.c was predicted to be homologous with motif-1 and motif-4 of the PROTEASOME signature sequence. However, the third fingerprint, MTPLANTPEC, provides signature sequences for metallothionein PEC superfamily members. The MTPLANTPEC is a five-element fingerprint derived from the initial alignment of six sequences. Motif-1 has three metal-binding cysteine residues; however, motifs 2 to 5 contain cysteine residues 1, 2, 4, and 3, respectively, for metal binding. The translated protein sequence of Ds-DbRCaG-01-Rga.c had shown a match with motif-2 and motif-5 of the MTPLANTPEC signature.

### 3.4 Annotation of protein secondary structure

The SOPMA and PSIPRED programs were used for secondary structure assessments. The α-helix (Hh), random coil (Cc), and extended strand (Ee) percentiles were observed in the translated protein sequence of *D. sissoo* RGAs. The type of amino acid (polar, non-polar, hydrophobic, and aromatic), annotation grid or sequence plot, and PSIPRED chart were also computed (**Supplementary Figure 1**). In Ds-DbRCaG-02-Rga.a, a significant share, 63.89%, of the protein secondary structure Ee was recorded, while the percentage of other shared structural features was 2.78% for Hh, 13.89% for Tt, and 19.44% for Cc. The translated protein sequence of Ds-DbRCaG-04-Rga.b predicted 22.8% of Hh, 31.58% of Ee, and 10.53% of Tt, with a significant Cc share of 35.09%. Likewise, the translated protein sequence of Ds-DbRCaG-06-Rga.c predicted 25.98% of Ee and 12.60% of Tt, with a considerable share, 61.4%, of Cc.

### 3.5 Homology modeling

Different online servers were used for the structural and functional characterization of the *D. sissoo* RGAs. The deduced amino acid sequences of the identified RGAs were subjected to SWISS-MODEL and PHYRE2 for template selection.

The Ds-DbRCaG-02-Rga.a showed maximum homology with a significant hit to template 3UAF (the crystal structure of a TTR-52 mutant of *Caenorhabditis elegans*). Twenty-three amino acid residues of Ds-DbRCaG-02-Rga.a (11-33) were modeled with template 3UAF residues (48-77) with 53.4% confidence and 39% sequence identity. The aligned region of the template confirmed the TTR52 superfamily (Transthyretin-like family) domain.

The Ds-DbRCaG-04-Rga.b showed homology with a significant hit to the template 2W27 (Crystal structure of the *Bacillus subtilis* YkuI protein an EAL domain, in complex with substrate c-di-GMP and calcium). Forty-six amino acid residues of Ds-DbRCaG-04-Rga.b (57-102) were modeled with template 2W27 residues (306-348) with 29.6% confidence and 21% sequence identity. However, the aligned region of the template confirmed EAL associated YkuI_C domain. This EAL domain is named after the presence of a conserved motif signature, Glutamate-Alanine-Leucine.

The Ds-DbRCaG-06-Rga.c showed homology with a significant hit to the template 3M1U (Crystal structure of a Putative gamma-D-glutamyl-L-diamino acid endopeptidase (DVU_0896) from *Desulfovibrio vulgaris* Hildenborough at 1.75 A resolution). Twenty-six amino acid residues (30-55) of Ds-DbRCaG-06-Rga.c were modeled with template 3M1U residues (135-160) with 62.6% confidence and 42% sequence identity. However, the template’s aligned region confirmed the SH3 (Src homology 3) domain, which involves regulating kinase activity.

### 3.6 3-D structural display

**Figure 2:**
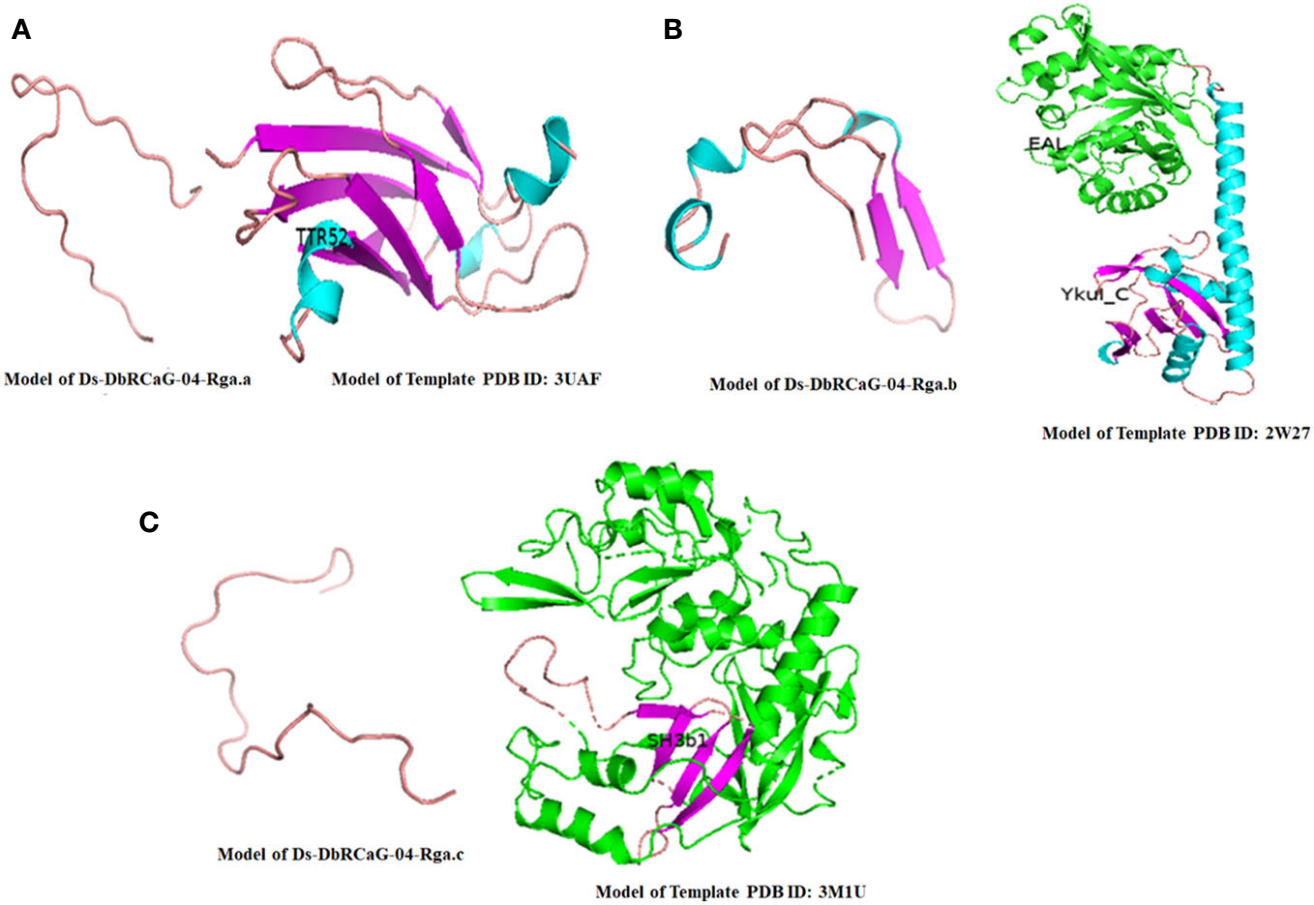
The 3-D model of *D sissoo* RGAs’ deduced amino acid sequences showing secondary structures with β-sheets represented in purple, α-helix in blue, and loops in salmon red color, along with their respective homology model (template) showing different domains in different colors. The identified domains of the *D sissoo* RGAs and their respective template domains are shown in the same colors. **(A)** the predicted protein structure of Ds-DbRCaG-02-Rga.a; **(B)** the predicted protein structure of Ds-DbRCaG-04-Rga.b; **(C)** the predicted protein structure of Ds-DbRCaG-06-Rga.c.

After template selection, 3D imaging of these models was generated using PyMOL v. 2.4.0 (**Figure 2**). The Ds-DbRCaG-02-Rga.a encoding protein showed structural homology to some parts of the TTR52 domain, consisting of a loop region, as shown in **Figure 2A**. The crystalline structure of template 2W27 predicted two domains: N-terminal EAL and C-terminal EAL, associated the YkuI_C domain, both connected with long α-helix. The Ds-DbRCaG-04-Rga.b encoding protein showed structural homology to some parts of the YkuI_C domain, typically containing six antiparallel β sheets packed against the helical structure, giving a pocket-like appearance. The crystalline structure of the Ds-DbRCaG-04-Rga.b encoding protein contained three small α-helix and two β-sheets joined via extended loops, as shown in **Figure 2B**. The crystalline structure of template 3M1U was predicted with four domains: N-terminal c-clip, SH3b1, SH3b2, and C-terminal NPLC-P60. However, the Ds-DbRCaG-06-Rga.c encoding protein showed structural homology to some regions of the SH3b1 domain. The SH3 has a β barrel structure mainly consisting of five or six β sheets. In contrast, the crystalline structure of Ds-DbRCaG-06-Rga.c encoding protein contained a loop region, as shown in **Figure 2C**.

### 3.7 3-D model validation

**Figure 3:**
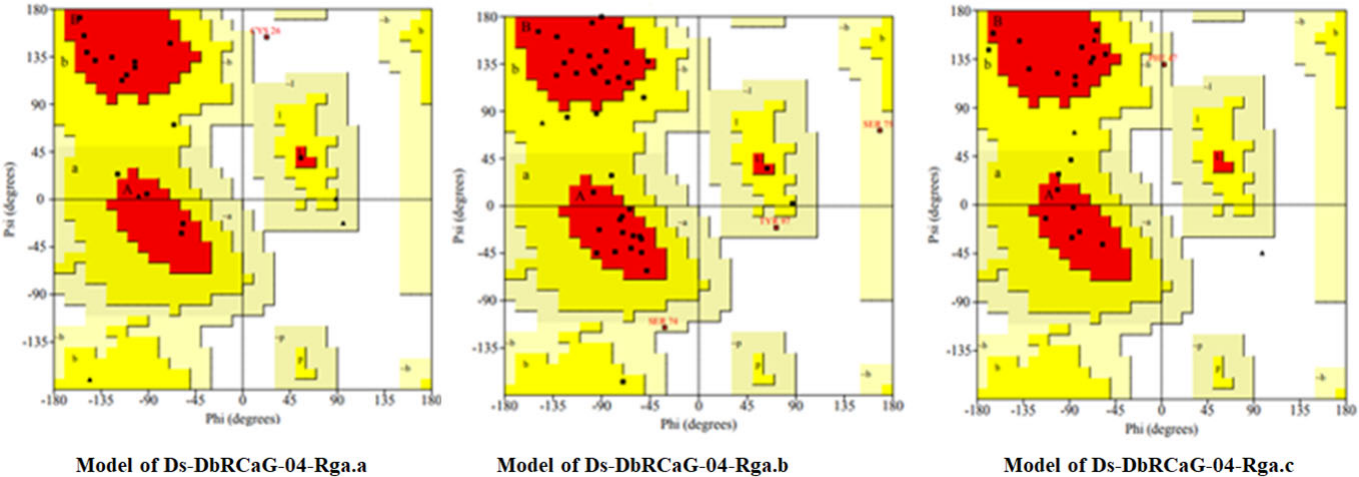
Ramachandran plot of translated protein sequences of *D. sissoo* RGAs Ds-DbRCaG-02-Rga.a, Ds-DbRCaG-04-Rga.b, and Ds-DbRCaG-06-Rga.c. The most favored region is in red, additionally allowed in yellow, generously allowed in light yellow, and disallowed region indicated in white fields.

**Figure 4:**
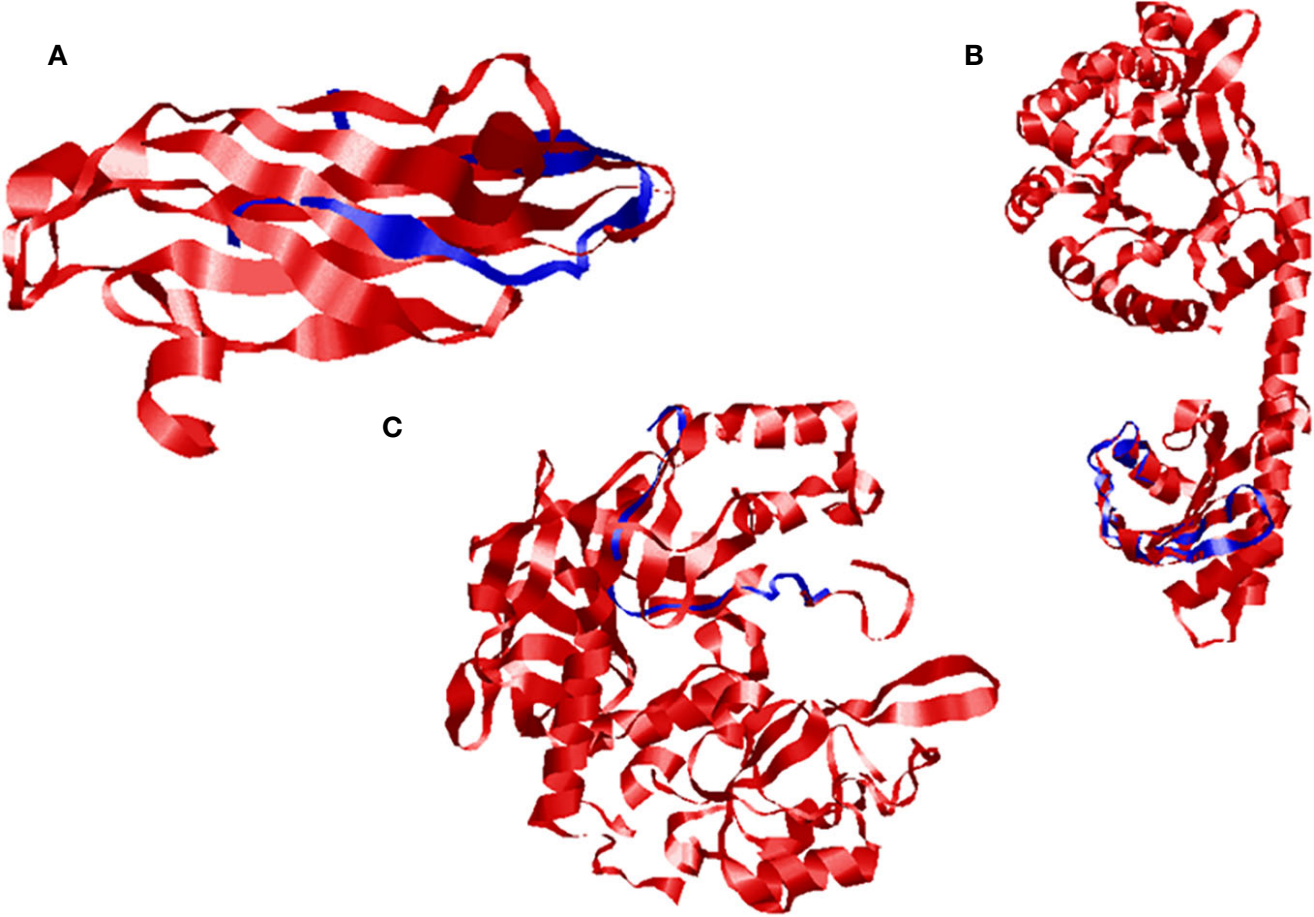
Superimposed ribbon structure of *D. sissoo* RGAs’ predicted protein with their respective template region. The blue indicates the *D. sissoo* RGA protein, while the red shows the template protein. **(A)** Ds-DbRCaG-02-Rga.a superposed to model 3UAF, **(B)** Ds-DbRCaG-04-Rga.b superposed to model 2W27, and **(C)** Ds-DbRCaG-06-Rga.c superposed to model 3M1U.

The quality was assessed by generating Ramachandran plots to confirm the reliability of homology-based generated 3D models. The model validation of Ds-DbRCaG-02-Rga.a predicted 82.4% residues in the most favored region and 11.8% in the additional allowed region with the remainder in the disallowed region. The model validation study of Ds-DbRCaG-04-Rga.b indicated 78.0% residues in the most favored region, 14.6% in the additional allowed region, and 7.3% in the generously allowed region. Similarly, a model validation study of Ds-DbRCaG-06-Rga.c indicated 83.3% residues in the most favored region, 11.1% in the additional allowed region, and 5.6% in the generously allowed region. Moreover, the calculated Z-scores for the predicted models of Ds-DbRCaG-02-Rga.a, Ds-DbRCaG-04-Rga.b, and Ds-DbRCaG-06-Rga.c were -0.16, -2.16, and -1.72, respectively, confirming the models to be satisfactory (**Figure 3**). The superimposition of the model with the template was also assessed along with the TM score and RMSD (root mean square deviation) values. The TM scores of Ds-DbRCaG-02-Rga.a, Ds-DbRCaG-04-Rga.b, and Ds-DbRCaG-06-Rga.c were 0.13608, 0.10621, and 0.05534, respectively, suggesting random structural similarity of these superimposed models with their template. While the RMSD values of Ds-DbRCaG-02-Rga.a, Ds-DbRCaG-04-Rga.b, and Ds-DbRCaG-06-Rga.c with their respective template were 2.44 Å, 0.73 Å, and 2.22 Å, respectively. The RMSD value scales the similarity among two atomic coordinates in a superimposed position. In the present findings, the measured RMSD values demonstrated that the residues were superimposed well (**Figure 4**). 

## 4 Discussion

NBS-LRR (class of R genes) based DOP-rtPCR analysis was performed to recover putative RGAs against dieback stress. In earlier studies, several plant species have been used in degenerate primers-based approaches to identify RGAs (Babar et al., 2021). These amplified *D. sissoo* RGAs were sequenced and characterized *in-silico* using a computational biology approach that proved part of the disease-resistance pathway. Subcellular localization confirms their location in the nuclear, mitochondrial, plasma membrane, and extracellular space. The extracellular location was also assessed with tobacco and Arabidopsis (Ijaz et al., 2022). The dynamic-related protein of rice (OsDRP1E) has a mitochondrial cellular location (Li et al., 2017) which defines their role in plants’ defense mechanism upon pathogenic attack. Similarly, AtPR1 proteins were confirmed with their localization in the plasma membrane (Pecenkova et al., 2017) and NPR1 in the nucleus (Kinkema et al., 2000).

Compartmentalization, surrounded by membranous structures, is characteristic of eukaryotic cells. These separate compartments specify distinct cellular functioning that is controlled by proteins. Thereby, subcellular localization prediction analysis was made in CELLO v 2.5, in which deduced amino acid sequences of identified RGAs of *D. sissoo* were submitted. It confirmed their role as a subcellular entity; among these, the extracellular location Ds-DbRCaG-02-Rga.a and Ds-DbRCaG-06-Rga.c (**Supplementary Table 2**) was similar to AtPR1of Arabidopsis (Pecenkova et al., 2017) and pathogenesis-related protein (PR proteins) of the tobacco plant with these being reported to be localized in extracellular space (Vigers et al., 1992). Similarly, *Plasmopara viticola* PR10.2 was identified to be localized in multiple subcellular compartments, i.e., cytoplasm, chloroplast, and nuclear region (He et al., 2013). However, SignalP predicted *D. sissoo* RGAs proteins’ role as secretory signal peptides (Sec/SP1 = Standard secretory signal peptides transported by the secretory peptidase 1-Lep). This predicted role is in accordance with the pathogen-related proteins (PR2a, PR2b, PR2c, PR2d) of *Nicotiana tabaccum* (Ward et al., 1991).

The biochemical and molecular functioning of proteins relies on these properties. Amino acid composition confirmed the low abundance of methionine (0.9-1.7%), tryptophan (1.6-1.8%), and aspartate (1.6-3.3%), while the higher abundance of leucine (10.5-15.3%), proline (10.5-13.3%), serine (11.9-15.0%) and arginine (8.3-16.7%) in the deduced amino acid sequences was similar to the result as described by Mohanta et al. (2019) for plant species.

The translated protein sequence of *D. sissoo* RGAs, when scanned for motif identification, displayed a protein kinase phosphorylation site that was observed in recovered RGAs. Various signaling pathways, including stress responses, mainly depend on protein kinases and phosphatases for phosphorylation or dephosphorylation (Sarwar et al., 2022). Similarly, salicylic acid (SA) activation-mediated defense-related genes pass through many phosphorylation events. For example, the casein kinase II (CK2) is a ser-thr protein kinase conserved to eukaryotes and regulates cellular processes (Ahmed et al., 2002) and was identified as an essential protein kinase that participated in the SA-activated pathway in the tobacco plant (Hidalgo et al., 2001).

The translated sequence of *D. sissoo* RGAs did not significantly hit the conserved domain available in NCBI’s conserved domain record. In protein fingerprinting search, these encoded proteins were predicted with protein fingerprints that were involved in stress response or immune response. The translated protein sequence of Ds-DbRCaG-02-Rga.a was predicted with ELONGATNFCT (elongation factor signature). The protein synthesis elongation factor (EF-Tu) binds to the GTP molecule during the translation process and is now thought to be involved in plant stress response. EF-Tu and EF-1α were associated with high-temperature stress (Fu et al., 2012).

The translated protein sequence of Ds-DbRCaG-04-Rga.b was predicted with a VDCCGAMMA6 (voltage-dependent Ca^2+^ channel gamma 6) fingerprint. Calcium ion (Ca^2+^) concentration is an essential indicator of signal transduction during plant growth, development, and stress. Voltage-dependent Ca^2+^ channels in the plasma membrane are responsible for Ca^2+^ influx and are activated upon ionic imbalance depolarization during hormonal, environmental, or pathogenic stimuli (Thuleau et al., 1994). These consist of four subunits: beta subunit (intracellular); gamma subunit (transmembrane); the cleaved product of the same gene, alpha-2; and delta subunit (Witcher et al., 1993).

The translated protein sequence of Ds-DbRCaG-06-Rga.c was predicted with three fingerprint sequences. TNFACTORR13C provides signature sequences for necrosis or apoptosis, used as an activated host defense (Chu, 2013). The second fingerprint is PROTEASOME and provides a signature for proteasome subunits. The protein misfolding, dysfunctioning, and malfunctioning due to plant stresses activate the proteolytic process or proteasome machinery that removes this damage to ensure the cell’s good physiological metabolism (Ali and Baek, 2020).

The third fingerprint, MTPLANTPEC, provides signature sequences for metallothionein PEC superfamily members. Metallothionein (MTs) and phytochelatins (PCs) proteins are involved in metal detoxification and also in stress response in plants (Shukla et al., 2016). During stress, MTs prevent plant cells from oxidative damage (Zhou et al., 2006). Previously, the MTs were induced upon viral infection in the tobacco plant (Choi et al., 1996).

The *in-silico* characterization of *D. sissoo* RGAs was performed using online web servers. Ds-DbRCaG-02-Rga.a showed significant homology with template PDB ID: 3UAF. The alignment region confirmed TTR52 (Transthyretin-52) domain acts as a bridging molecule for phagocytes and apoptotic cells, thus regulating immune responses, previously shown to regulate cold stress in Arabidopsis (Chen et al., 2020). The 3D model of the template predicted with the TTR52 domain typically consisted of seven β strands and two helices. However, the Ds-DbRCaG-02-Rga.a encoding protein showed structural homology with the loop region. Furthermore, Gly13, Phe15, Gly19, Ser22, Asp24, Leu27, His31, and Cys33 residues were conserved to template residues Gly50, Phe52, Gly56, Ser59, Asp68, Leu71, His75, and Cys77. Kang et al. (2012) explained that the N-terminus site’s loop regions (L2 and L3) work as active centers performing hydrophobic interaction with other molecules. The hydrophobic residues Phe15, Leu16, Leu17, Leu27, Ile28, Phe29, and Met32 were observed in Ds-DbRCaG-02-Rga.a and might be involved in hydrophobic interaction with the substrate molecule.

Ds-DbRCaG-02-Rga.a showed maximum homology with a significant hit to template 3UAF. The aligned region of the template confirmed the TTR52 superfamily (Transthyretin-like family) domain. It acts as a bridging molecule for phagocytes and apoptotic cells, thus regulating immune responses (Eletsky et al., 2012). In addition, TTL (Transthyretin-like) has been identified in Arabidopsis to regulate cold tolerance (Chen et al., 2020).

The *Ds-DbRCaG-04-Rga.b* showed homology with a significant hit to the template 2W27, and the aligned region of the template confirmed the EAL-associated YkuI_C domain. This EAL domain is named after the presence of a conserved motif signature, Glutamate-Alanine-Leucine. It possesses type-I cyclic-dimeric-GMP specific phosphodiesterase (PDE) activity and has been identified as a signaling protein that plays a role in infection (Dow et al., 2006).

The *Ds-DbRCaG-06-Rga.c* showed homology with a significant hit to the template 3M1U. The template’s aligned region confirmed the SH3 (Src homology 3) domain, which regulates kinase activity. The SH3 domain works as a polypeptide cofactor for activating or inhibiting different kinases (Shen et al., 2012). The SH3 domain mainly observed the part of signaling pathway proteins. In plants, the SH3 protein family is identified by three members; of these, SH3P1 and SH3P3 have been reported to be involved in cellular trafficking. In comparison, SH3P2 has been identified as being involved in plants’ autophagosome formation. Autophagy is a well-known mechanism for maintaining cellular homeostasis via the digestion and recycling of cellular compounds, including the degradation of dysfunctional molecules (proteins, protein aggregates, and lipids) or damaged organelles and regulating plant immune response to external stimuli.

## Data availability statement

The original contributions presented in the study are included in the article/**Supplementary Material**, further inquiries can be directed to the corresponding author.

## Author contributions

SI and IU conceived, designed, and developed the experiment. SI, IU, HR, and BN experimented. SI and IU analyzed and interpreted the data. SI, IU, HR, BN, HA, and SK drafted and revised the manuscript. All authors contributed to the article and approved the submitted version.
