# Supplementary material for: In silico structural-functional characterization of three differentially expressed resistance gene analogs identified in Dalbergia sissoo against dieback disease reveals their role in immune response regulation
Source: Front Plant Sci. 2023 Oct 16;14:1134806. doi: 10.3389/fpls.2023.1134806 (PMC10613980; doi:10.3389/fpls.2023.1134806)
Supplement: Supplementary file 1 [file DataSheet_1.docx]

**Table S1:** Degenerate primers of NBS domain used to recover putative resistance gene sequences (RGAs) upregulated against dieback disease in shisham.

| *Degenerate primer* | *Primer sequence (5′-3′)* | *References* |
| --- | --- | --- |
| dgPL-a1F | GGNGGNRTNGGNAAGACGAC | (Noir *et al*., 2001) |
| dgPL-a2F | GGNGGNRTIGGIAARACIAC | (Sun *et al*., 2010) |
| dgPL-a3F | GGIGGIGTIGGIAAIACIAC | (Leister *et al*., 1996) |
| dgPL-a4F | TGSSRGGHWYRGGBAAAACTAC | (Zhang *et al*., 2008) |
| dgPL-a5F | GGTGGGGTTGGGAAGACAACG | (Leister *et al*., 1996) |
| dgGL-b1R | GAGGGCTAAAGGAAGGCC | (Deng *et al*., 2000) |
| dgGL-b2R | IAGIGCIAGIGGIAGICC | (Leister *et al*., 1996) |
| dgGL-b3R | AAGIGCTAAGIGGIAAGICC | (Peraza-Echeverria *et al*., 2008) |
| dgGL-b4R | HRCWARAGGVARCCCTYBACA | **(**Naresh *et al*., 2017). |
| dgGL-b5R | GAGGGCNARNGGNAAICC | (Noir *et al*., 2001) |

| **Name** | **Protein length** | **Mw (Da)** | **Molecular formula** | **Theoretical (pI)** | **Instability index** (**II)** | **Aliphatic index** | **GRAVY** | **Subcellular location** |
| --- | --- | --- | --- | --- | --- | --- | --- | --- |
| Ds-DbRCaG-02-Rga.a | 36 | 3897.60 | C_172_H_275_N_47_O_48_S_4_ | 8.89 | 37.87 | 97.50 | 0.675 | Extracellular |
|  |  |  |  |  |  |  |  | Mitochondrial |
| Ds-DbRCaG-04-Rga.b | 114 | 13241.32 | C_603_H_950_N_168_O_164_S_2_ | 9.72 | 40.41 | 104.21 | -0.121 | Plasma membrane |
|  |  |  |  |  |  |  |  | Nuclear |
| Ds-DbRCaG-06-Rga.c | 127 | 13417.33 | C_606_H_942_N_166_O_173_S_3_ | 10.03 | 50.09 | 70.63 | -0.173 | Extracellular |
|  |  |  |  |  |  |  |  | Nuclear |

**Supplementary Table 2: Subcellular localization and physicochemical parameters of *D. sissoo* RGAs collected under dieback challenge**


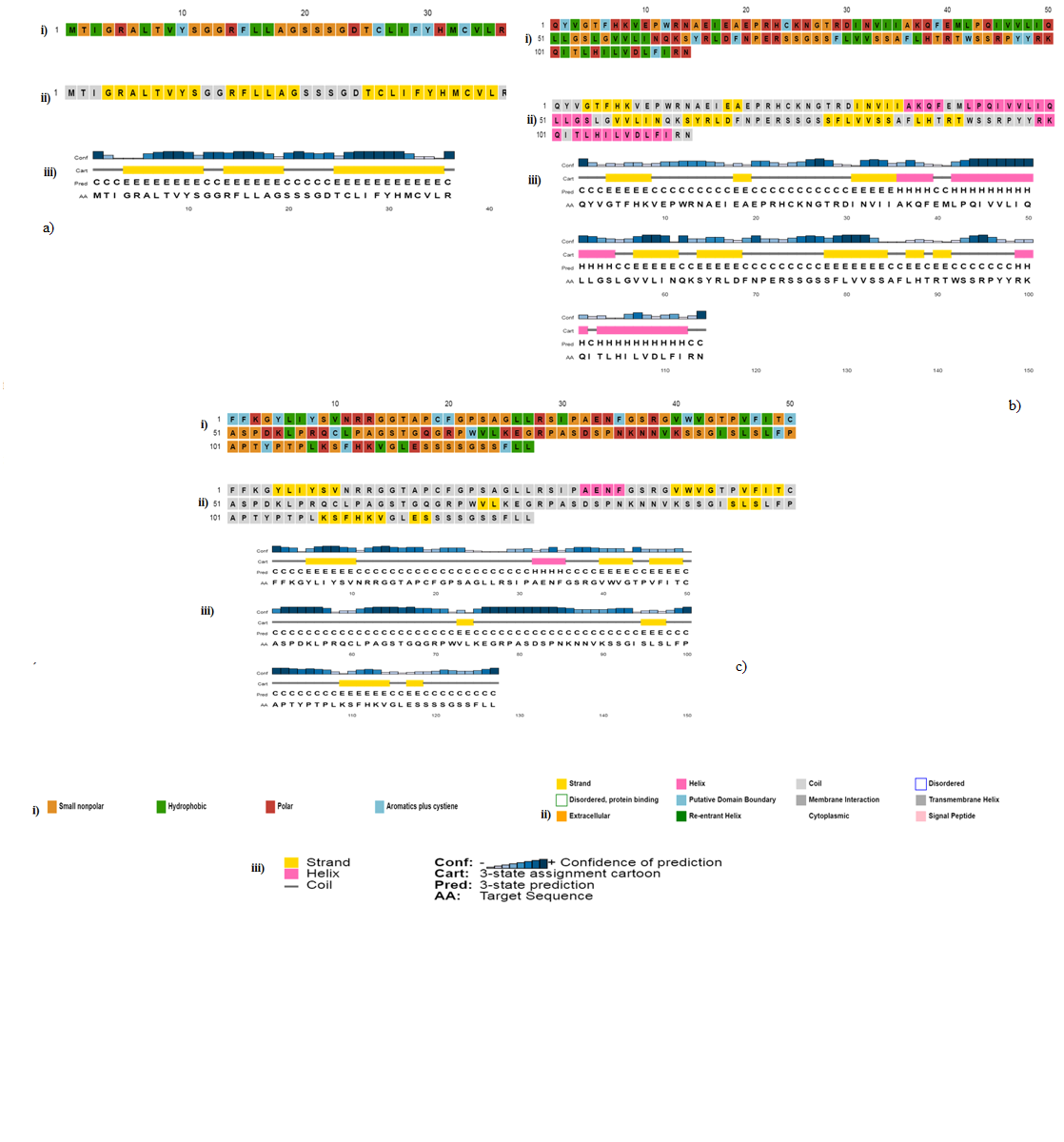


**Supplementary Figure 1:** Predicted secondary structure of *D. sissoo* RGAs showing **i)** the amino acid type (polar, non-polar, hydrophobic, and aromatic), **ii)** annotation grid or sequence plot, and **iii)** PSIPRED chart. **a)** Ds-DbRCaG-02-Rga.a, **b)** Ds-DbRCaG-04-Rga.b, **c)** Ds-DbRCaG-06-Rga.c
